# Effect of low-dose amitriptyline on reducing pain in clinical knee osteoarthritis compared to benztropine: study protocol of a randomised, double blind, placebo-controlled trial

**DOI:** 10.1186/s12891-021-04690-y

**Published:** 2021-09-27

**Authors:** Anita E. Wluka, Donna M. Urquhart, Andrew J. Teichtahl, Sultana Monira Hussain, Andrew Forbes, Carolyn Arnold, Yuanyuan Wang, Flavia M. Cicuttini

**Affiliations:** 1 Department of Epidemiology and Preventive Medicine, School of Public Health and Preventive Medicine, Monash University, Melbourne, Victoria Australia; 2 Alfred Health, Commercial Road, Melbourne, Victoria Australia

**Keywords:** Knee osteoarthritis, Pain, Amitriptyline

## Abstract

**Background:**

Knee osteoarthritis is a major cause of pain and disability. Pain control is poor, with most patients remaining in moderate to severe pain. This may be because central causes of pain, a common contributor to knee pain, are not affected by current treatment strategies. Antidepressants, such as amitriptyline, have been used to treat chronic pain in other conditions. The aim of this randomised, double blind, controlled trial, is to determine whether low dose amitriptyline reduces pain in people with painful knee osteoarthritis over 3 months compared to benztropine, an active placebo.

**Methods/design:**

One hundred and sixty people with painful radiographic knee osteoarthritis will be recruited via clinicians, local and social media advertising. Participants will be randomly allocated in a 1:1 ratio to receive either low dose amitriptyline (25 mg) or active placebo (benztropine mesylate, 1 mg) for 3 months. The primary outcome is change from baseline in knee pain (WOMAC pain subscale) at 12 weeks. Secondary outcomes include change in function (total WOMAC) and the proportion of individuals achieving a substantial response (≥ 50% reduction in pain intensity, measured by Visual Analog Scale, VAS, from no pain to worst pain imaginable, 0-100 mm) and moderate response (≥ 30% reduction in pain intensity, measured by VAS) at 12 weeks. Intention to treat analyses will be performed. Subgroup analyses will be done.

**Discussion:**

This study will provide high level evidence regarding the effectiveness of low dose amitriptyline compared to benztropine in reducing pain and improving function in knee OA. This trial has the potential to provide an effective new therapeutic approach for pain management in knee osteoarthritis, with the potential of ready translation into clinical practice, as it is repurposing an old drug, which is familiar to clinicians and with a well described safety record.

**Trial registration:**

Australian New Zealand Clinical Trials Registry prior to recruitment commencing (ACTRN12615000301561, March 31, 2015, amended 14 December 2018, February 2021). Additional amendment requested 18 July 2021.

## Background

Knee osteoarthritis (OA) is a major cause of pain and disability, estimated to affect 654 million people worldwide [1], with the lifetime risk estimated to be 44.7% in the USA [2]. Most people with OA (58%) live with moderate to severe chronic pain [3]. People with knee OA rank pain as their highest treatment priority [4], as it limits function, independence, quality of life and leads to costly joint replacement [5].

The management of knee OA aims to reduce pain, optimise function and reduce disease progression. OA management guidelines focus on patient education, weight management and exercise therapy [6], but even with excellent patient engagement, the effect sizes of these interventions are moderate at best [7]. Practically, pharmacological interventions are the mainstay of pain control. However, their effect is also limited. A recent Cochrane review concluded that paracetamol provides only minimal clinical benefit [8]. Although non-steroidal anti-inflammatory drugs (NSAIDs) provide a small to moderate effect on analgesia, they are associated with significant adverse effects, particularly in the elderly which limits their use [9]. Although opiates are used [6], with no proven long term benefit [10], there is evidence of significant harm [11]. As a result, over half (54%) of patients with knee OA report that their knee pain is not well controlled [12].

Pain control in OA may fail because treatment is not targeted to the cause of pain which is multifactorial. Although the severity of structural pathology is associated with the prevalence of pain, it does not explain the intensity of pain [13, 14], suggesting that factors outside the joint play a role. Pain sensitisation, is an abnormal state of responsiveness or increased gain in the reception of pain [15]. Pain sensitisation may play a role in some patients with OA, where a painful peripheral stimulus relating to the joint is amplified and augmented by changes in the spinal cord and central nervous system [15]. Clarifying whether this is the cause of pain in an individual is important, as response to therapy will be influenced by the cause of pain [16].

For pain related to sensitisation, the most commonly used analgesics in OA, paracetamol, COX2 inhibitors, NSAIDs and opioids may be ineffective [16]. For central causes of pain and sensitisation, antidepressants may be helpful. Duloxetine, a serotonin and norepinephrine reuptake inhibitor (SNRI) antidepressant, has been conditionally recommended for use by international OA guidelines for some people with OA, although which patients are not specified [6]. Low dose tricyclic antidepressants, are commonly used in chronic neuropathic pain states (eg diabetic peripheral neuropathy [17]) and central pain states (eg fibromyalgia [17]). They have not been fully trialled in OA [17]. Until recently, human studies of low dose tricyclic antidepressants for knee pain in OA have been limited to 2 small studies; although they showed improvement in knee pain they had no control population [18, 19]. There has only been a single recent controlled trial of nortriptyline, but not amitriptyline, in patients with painful clinical knee OA [20]: radiographic criteria were not used.

### Hypothesis and objectives

We aim to determine whether the use of low dose amitriptyline reduces pain in people with painful radiographic knee OA over 3 months compared to the use of benztropine, an active placebo. Our secondary objectives include determining whether amitriptyline improves knee function and results in a higher proportion of people achieving a clinically significant reduction in pain compared to those on placebo. We hypothesis that those taking low dose amitriptyline will have improved pain and function compared to those taking active placebo, and that the proportion achieving a clinically significant improvement will be higher in participants receiving amitriptyline compared to those on placebo.

## Methods

### Study design

The Knee Osteoarthritis Pain Study (KOPS) is a single centre, pragmatic, randomised, double blind, active placebo controlled clinical trial over 12 weeks.

### Trial registration

The trial was registered on the Australian New Zealand Clinical Trials Registry prior to recruitment commencing (ACTRN12615000301561, March 31, 2015, amended 14 December 2018, February 2021). Reporting will be in accordance with the Consolidated Standards of Reporting Trials (CONSORT) Statement [21]. Protocol reporting is in accordance with the SPIRIT Statement (Standard Protocol Items: Recommendations for Interventional Trials) [22]. In December 2018, the protocol was amended to include participants with severe radiographic knee OA (grade 3 according to OA Research Society International atlas) on the basis of pain sensitisation, the mechanism targeted by amitriptyline, being prevalent in approximately 30% of those with more severe disease [23]. The protocol was further amended in February 2021 to remove the OARSI responder criteria as a secondary outcome on the basis of uncollected data [24, 25], with replacement by the proportion achieving a substantial and moderate response, as defined by the Initiative on Methods, Measurement, and Pain Assessment in Clinical Trials (IMMPACT), in July 2021 [24].

### Ethics approval

Ethics approval has been obtained from the Alfred Hospital Ethics Committee (512/14) and registered with the Monash University Human Research and Ethics Committee (CF15/1488–2,015,000,729). Participants will only be included after they have provided informed consent.

### Study setting and participants

In a single site study, we aim to recruit 160 participants from the Victorian community with painful knee OA. Recruitment will use a combined strategy involving general practitioners, rheumatologists, orthopaedic surgeons, and advertising in local and social media.

### Eligibility criteria

#### Inclusion criteria

Male and female participants with painful knee OA (pain for ≥ 3 months), defined according to ACR clinical and radiographic criteria, with pain of > 30 mm on a 100 mm visual analogue scale (VAS) and age 40–75 years required.

#### Exclusion criteria

These include: 1) Inability to give informed consent; 2) Intra articular therapy over the past 3 months; planned injection or surgical intervention in the next 4 months 3) Rheumatoid or other inflammatory arthritis, or significant knee injury; 4) Major depressive disorder for which anti-depressant therapy is indicated 5) Taking medications that are contra-indicated when taking amitriptyline (monoamine oxidase inhibitors, other antidepressants, opioids, drugs that inhibit CYP3A4, etc) 6) Co-morbidity that may limit participation (eg. planned joint replacement in the next 4 months, medical conditions eg. malignancy in the past 5 years other than non-melanoma skin cancer) or relocation; 7) Fibromyalgia, as this may provide an alternative underlying explanatory condition 8) Contraindication to amitriptyline eg acute angle glaucoma, prostatism, etc. 9) Pregnancy or planned pregnancy. Severe radiographic knee OA (grade 3 according to OA Research Society International atlas) was initially a cause for exclusion, which was amended in the protocol in December 2018 as pain sensitisation, the mechanism targeted by amitriptyline, is prevalent in those with more severe disease [23].

### Participant timeline

**Fig. 1:**
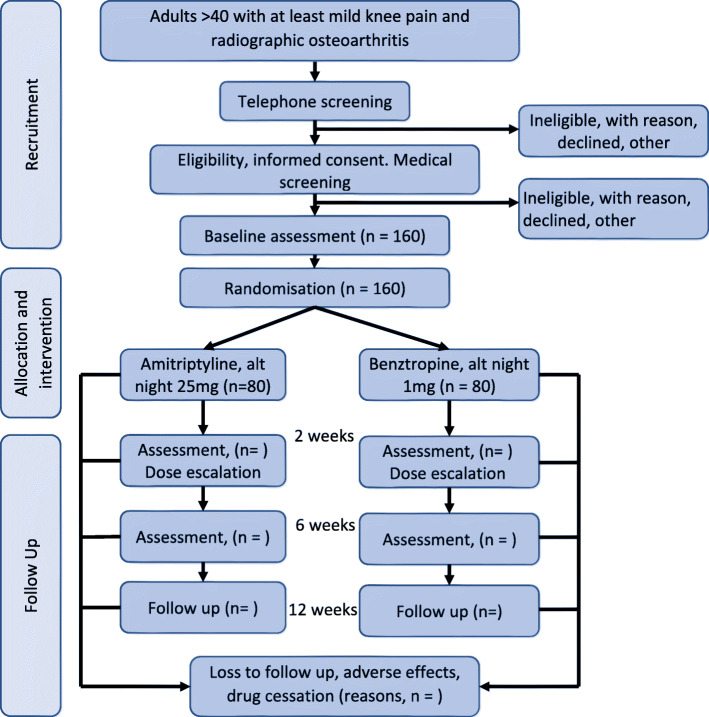
Flow of participants

Table 1 shows the assessments at each time point, according to the Standard Protocol Items: Recommendations for Interventional Trials (SPIRIT Statement). Figure 1 provides the flow of participants in the study.

**Table 1:** Schedule of study flow and assessments

	Enrolment	Baseline	2 weeks	6 weeks	12 weeks
**ENROLMENT:**					
Eligibility screen	x				
Informed consent	x				
Clinical assessment		x			
Randomisation		x			
**INTERVENTION:**					
Amitriptyline, 25 mg nocte		Alternate	Nightly	Nightly	Nightly
Benztropine, 1 mg, nocte		Alternate	Nightly	Nightly	Nightly
**ASSESSMENTS**					
**Primary and secondary outcomes**					
WOMAC (pain, stiffness, function, total)		x			x
**Other measures of knee pain**					
Pain severity (NRS, 0–10)		x	x	x	x
Pain severity (VAS, 100 mm), pain diagram		x			x
PainDETECT		x			x
**Functional status and severity**					
ACR functional class I, II or III		x			x
Investigator assessed global assessment of OA status (Likert, 0 very well–4 very poor)		x			x
General health status (AQOL-8D)		x			x
Leg muscle strength (in triplicate)		x			x
Xray – KL score, osteophytes, JSN		x			
**Other measures of response**					
Pain acceptability (48 h)		x			x
Patient global impression of change (Likert, 0–7)			x	x	x
**Other measures**					
Inclusion criteria: pain & 1 of age > 50, stiff <30mins, Crepitus, osteophytes and KOA		x			
Age, height, weight, bp, smoking		x			
Medical conditions		x			x
Education (category), Employment current status, type of work (manual, office, NA)		x			
Medications – analgesics, NSAID, opioid		x			x
Fear of movement (TSK)		x			x
Mood disorder (HADS)		x			x
Physical activity (IPAQ, 7 days)		x			x
Sleep quality and disturbance (PSQI)		x			x
**Treatment and safety issues**					
Medications – analgesics, NSAID, opioid		x			x
Treatment belief: study drug or placebo					x
Rating of medication and side effects (Scale, excellent, good, fair, poor, unacceptable)					x
UKU		x	x	x	x
Adverse events			x	x	x
Adherence, pill count					x

*WOMAC* Western Ontario and McMaster Arthritis Index. *NRS* Numerical rating scale *VAS* visual analogue scale, *AQOL-8D* Assessment of quality of life. *TSK* Tampa Scale of Kinesiophobia *HADS* Hospital anxiety and depression scale *IPAQ* International physical activity questionnaire *PSQI* Pittsburgh sleep quality index UKU: UKU Side effect rating scale for psychotropic drugs

### Intervention

Participants will be randomised to treatment with capsules, identical in appearance, containing either 1) low-dose amitriptyline (Symbion, NSW), initially 25 mg on alternate days for the first 2 weeks, to improve compliance and reduce side effects, increased to 25 mg daily thereafter; or 2) benztropine, an active placebo, at a dose of 1 mg alternate days for the first 2 weeks, to improve compliance and reduce side effects, increased to 1 mg daily thereafter (Symbion, USA).

Amitriptyline was chosen, being the most effective tricyclic antidepressant for managing neuropathic pain and fibromyalgia, at a dose used in these conditions [17]. Benztropine, an active placebo, was chosen as it mimics the anti-cholinergic effects of amitriptyline (eg dry mouth, constipation), and is not known to effect pain. Benztropine will produce more effective blinding of study participants and investigators than an inert placebo. The reduction in potential for unblinding explains why benztropine has been used as an active placebo in previous trials [26].

### Randomisation, allocation concealment, double blinding and control of bias

Participants will be randomised in a 1:1 ratio, to amitriptyline or benztropine, according to a computer-generated randomisation sequence with permuted blocks of random sizes, held securely by the dispensing clinical trials pharmacy. This will be generated by a statistician, with no direct involvement in study execution. This process ensures that participant allocation cannot be accessed or influenced by research personnel.

The randomised controlled trial, will be double blind, with both participants and investigators assessing study outcomes blinded to treatment allocation. Allocation concealment and double blinding will be achieved by 1) dispensing by clinical trials pharmacy; 2) using an active placebo, identical in appearance to active study drug, with side effects that mimic those of amitriptyline with dose escalation; 3) subjective measures of study outcomes being taken by research assistants uninvolved in other aspects of the trial and unaware of group allocation; 4) data analysis will be completed prior to the allocation code being revealed.

Unblinding will be possible in the event of medical necessity, impacting on study participant safety. Code-break envelopes for the randomisation schedule will be held by Monash University. Should participants be unblinded, they will be withdrawn from the trial.

### Study procedure and participant timeline

Potential participants will be screened by telephone to determine eligibility. They will be provided trial information and invited for clinical assessment (either face to face, or via Zoom, during lockdown due to the COVID-19 pandemic). Consent obtained by study physician. At clinical screening, the participant will complete questionnaires, and be assessed against inclusion and exclusion criteria for participation. The study knee will be the one with clinical and radiographic OA, meeting eligibility criteria. Where both knees are symptomatic, the radiographically less severe knee will be used. Where the radiographic severity is the same, the knee with the higher level of pain will be used. The study scheduled assessments and measures are presented in Table 1. At the 4 participant contacts, safety and drug efficacy will be assessed. Participant receipt of study drug will be confirmed within 3 days of prescription.

Throughout the study period, all other usual therapy will be allowed, with the exception of medications that may interact with study medication.

#### Quality assurance

Study personnel will be trained according to the study protocol. They will execute the study using the study’s standard operating protocol book, detailing the procedures to be used, study contacts, measures. Participant assessments will be collected in pre-specified case report forms.

Pill count at the end of the study period will be used to assess compliance with study medication. Participants will be reimbursed for parking and transport costs: they will not be paid to participate in the trial.

### Outcome measures

#### Primary outcome

Change in knee pain between baseline and 12 weeks will be assessed using the Western Ontario and McMaster Universities Osteoarthritis Index (WOMAC) pain scale, a self-administered questionnaire using VAS [27, 28]. Each of the 5 pain scale items will be assessed on a 100 mm VAS. Results will be summed and normalised to a 0–100 scale. Incomplete items will be addressed as recommended in the users guide: where one item is missing, the remainder will be averaged and divided by 5 then normalised. When more than one item is missing, the data will be deemed invalid [28].

#### Secondary outcomes

Change in knee symptomatology between baseline and 12 weeks will be assessed using *change in total WOMAC* score (5 pain items, 2 stiffness items and 17 function items, each measured on a 100 mm VAS will be added and the results normalised to 1–100). A measure of knee overall function is recommended by OARSI.

*Pain severity, achieving a clinically significant response* assessed by percentage reduction in participant’s current level of knee pain, recorded on VAS, ranging from no pain to the worst pain imaginable (0 – 100 mm), from baseline to 12 weeks. The proportion attaining a substantial response (designated ≥ 50% reduction in pain intensity) and a moderate response (designated as a ≥ 30% reduction in pain intensity, as defined by the International Pain Society [24, 25].

### Other measures

#### Measures of knee pain

*Pain severity* (Numerical Rating Scale, NRS) will be assessed at each participant contact, including telephone contacts (0, 2, 6, 12 weeks) using a categorised numerical rating scale (0–10). (How would you rate your pain on a 0–10 scale at the present time? 0 is no pain, 10 is pain as bad as it could be). This will be used for an area under the curve analysis, and not for assessment of change between 0 and 12 weeks.

*Pain diagram:* participants will indicate body areas where they feel pain at 0 and 12 weeks.

*Pain Detect* questionnaire will be applied at 0 and 12 weeks. (0–35). Scores of > 12 indicate increased odds of central pain sensitisation in OA [29].

#### Measures of functional status and disease severity

*ACR functional class rating* [30] *will be assessed*) at baseline and 12 weeks using Likert scale 0–4.

*Investigator assessed global assessment* of OA (Likert scale, 0–4) at baseline and 12 weeks.

*General health status* at 0 and 12 weeks, quality adjusted life years (QALYS) will be derived from the generic preference based, validated Assessment of Quality of Life 8D (AQol-8D) measure [31, 32].

*Muscle strength,* predominantly of quadriceps and hip flexors, measured by dynamometry at both legs simultaneously at baseline and 12 weeks [33].

*Severity of radiographic knee OA:* Osteophytes and joint space narrowing will be graded from a standing semi-flexed AP radiograph of the study knee using the Altman atlas (0–3, [34]). We have high reproducibility for grading at the knee (Kappa statistic 0.93, [35]).

#### Other measures of response

*Pain acceptability,* over the past 48 h at baseline and 12 weeks. (Thinking only of the pain you felt in your knee during the last 48 h, if you were to remain with that pain for the rest of your life would that be acceptable to you?)

*Patient’s global impression of change scale*, measured on Likert scale, 0–7 at 2, 6 and 12 weeks. (Since joining the study, would you describe the change (if any) in activity, limitations, symptoms, emotions and overall quality of life, related to your painful condition (knee pain)?)

#### Other measures

*Demographic and anthropomorphic measures*: Age, gender, height, weight, smoking, medical conditions, highest education level, current employment and current medications will be measured at baseline.

*Tampa Scale of Kinesiophobia (TSK) Scale (17 item)* will be used to assess fear of movement/(re) injury, at 0 and 12 weeks.

*International physical activity questionnaire (IPAQ)-SF* [36] at 0 and 12 weeks will assess physical activity.

*Pittsburgh sleep quality index* (PSQI) [37], administered at 0 and 12 weeks to assess sleep quality and disturbance.

*Hospital Anxiety and Depression Scale* [38]: Mood symptom severity (anxiety and depression subscales) will be assessed at 0 and 12 weeks.

#### Treatment and safety issues

*Analgesic use* will be documented at 0 and 12 weeks.

*Treatment guessing* at 12 weeks participants will be asked to guess whether they were on active study drug, placebo or whether they were not sure.

*Participant rating of study drug and side effects* on a likert scale, ranging from excellent to unacceptable.

*Adherence and compliance:* Participants will return unused study drug at the final visit. Capsules will be counted to assess adherence.

### Safety monitoring

*The UKU Side Effects Rating Scale* [39], a standardised, physician-administered interview for assessing the severity and impact of side effects of psychotropic drugs on daily function will be used to assess for adverse effects. In addition, participants will be asked to contact research staff in the event of any adverse events. Any serious adverse events and their relationship to study medication will be recorded and reported to the Ethics Committees. Participants’ physicians will be informed of their patient’s involvement in the study.

*Adverse events* will be recorded where present, by participant report on their occurrence and actively elicited at each contact. Should adverse events occur, participants will be followed until these have resolved.

### Dissemination policy

Trial results, regardless of statistical significance, will be presented in the peer reviewed literature after the final participant completes the study period. All authors will be required to contribute to and approve the submitted manuscripts, as per the Vancouver authorship guidelines. Upon publication of the primary manuscript, participants will be provided with the results, and informed of their group allocation.

### Sample size

#### Primary outcome

Based on published data, in OA, pain on the WOMAC pain scale (0-100 mm) has a standard deviation (SD) of ≈20 mm [40]. The minimal clinically important difference is 10 mm, with an expectation that 1 in 2 will experience substantial pain relief [41].

This outcome will be analysed using analysis of covariance (ANCOVA) of change in WOMAC pain adjusting for baseline pain. With 64 participants in each arm we will have 80% power to detect a 10 mm difference in change in pain between the intervention and control groups (alpha error 0.05, 2-sided significance at 12 weeks), conservatively assuming a pre-post correlation of zero. With positive correlation the power will be greater than 80%. Based on a conservative 20% loss to follow up we will recruit 160 participants (80 in each arm of the study).

#### Secondary outcomes

The detectable differences of secondary outcomes that will be able to be detected with an alpha error of 0.05, 2-sided significance and 80% power are as follows:

1. *Difference in WOMAC total* of 10 mm, normalised 0-100 mm, with a SD of 20 mm.
2. *Difference in proportion of participants achieving a 30 and 50% reduction in pain (VAS) at 12 weeks of 21.7%* based on the published placebo response in pooled results from 2 clinical trials of duloxetine in knee OA of 44.9 and 30.9% respectively [42].

### Statistical analysis

Summary statistics comparing randomised arms at baseline will be tabulated. Intention to treat analyses of primary and secondary continuous outcomes will be performed by linear regression adjusting for the baseline of the outcome variable where relevant. The proportion achieving each of ≥ 30 and ≥ 50% reduction in pain, representing “moderate improvement” and “substantial improvement” will be examined using log-binomial regression to estimate risk ratios and confidence intervals directly, as will other binary outcomes. Ordinal outcomes will use proportional odds regression models, with assessment of the proportionality. If the proportion of missing data in an outcome is greater than 5% then multiple imputation will be used. Supplementary analyses will be performed adjusting for imbalanced baseline factors prognostic of outcome. The total quantum of pain for each subject will use the area under the curve of the NRS over 0, 2, 6 and 12 weeks, with analysis performed using linear regression.

***Prespecified sub group analyses*** will be performed according to gender, median age, the painDETECT score > 12 or < =12 (central pain sensitisation), TSK scale > 37 or ≤ 37 (fear avoidance), PSQI > 5 vs PSQI ≤ 5 (sleep quality), HADS-A ≥ 8 vs < 8 (Anxiety), HADS-D ≥ 8 vs < 8 (Depression), HADS-A or HADS-D ≥ 8 or < 8 8 (Mood disorder), in those with and without previous injury, previous knee surgery, and according to radiographic grade of osteoarthritis (define categories/grades). Analyses will use regression models with interaction terms for intervention with each subgroup factor.

No adjustment will be made for multiple testing and statistical significance will be set as a two-sided *p* value < 0.05.

### Data integrity and management

Data will be collected using prespecified, case report forms. These will be securely stored within School of Public Health and Preventive Medicine, Monash University, with restricted access. Data will be entered in duplicate and stored electronically on a password protected server, with secure restricted access. Daily back up offsite will occur to minimise any loss of entered data. Study data will remain strictly confidential. Data transfer will only occur using deidentified data. Access will be limited to study personnel.

Following study completion, case report forms will be securely archived. Identifiers will not be removed in the case participants will require follow up in future. Electronic data will be held securely, on the secure server, password protected and accessible only to study investigators.

### Withdrawal and drug discontinuation

Should participants withdraw from the study or discontinue study drug prior to 3 months, the reason and date will be recorded. They will be requested to provide the remaining outcome measures.

### Monitoring

Monitoring of the study execution will be performed by the study investigators. They will ensure that trial execution is consistent with the study protocol. Regular meetings will ensure efficient study completion and monitoring of adverse events. Investigators will perform annual self-auditing of study execution according to the School of Public Health and Preventive Medicine, Monash University.

## Discussion

We propose a single centre, randomised, double blind controlled clinical trial to determine whether the use of amitriptyline reduces knee pain, function and provides overall benefit in people with knee OA. We will examine both efficacy and safety of this therapy.

Given that pain is heterogeneous in OA, we will examine the effect of amitriptyline in clinically defined subgroups (eg pain severity, pain sensitisation, fear avoidance, poor sleep, mood disorders, radiographic change, etc) to determine whether amitriptyline is more effective in certain patient phenotypes. If amitriptyline is found to be effective, then it will provide high quality evidence for a new avenue of therapy for use in knee OA, with the potential to improve pain management in patients with knee OA.

There is an urgent need for effective pain management strategies in knee OA, as over half of those with knee OA have unsatisfactory pain control [43]. To address this clinical problem, treatments targeted to alternative mechanisms of pain in knee OA, such as sensitisation, have been sought. Antidepressants have been used for many years in the management of low back pain. However, only the use of duloxetine, an SNRI, has been tested in a number of clinical trials to treat pain in knee OA [44]. Although a recent meta-analysis found low certainty evidence of a reduction in pain, it did not examine the proportion achieving a clinically important improvement [44]. Other previous analyses have suggested the proportion of patients achieving a clinically significant improvement is higher in treated participants [45]. A single recent study of low dose nortriptyline, a tricyclic antidepressant, found no effect overall [20]. However, this study recruited patients with clinically diagnosed knee OA, and did not have radiographic entry criteria. It did not use an active placebo which may explain why there were significant differences in anticholinergic side effects in those on study drug and placebo. Although there was not a significant improvement in pain, there was a tendency for improvement with a those on nortriptyline having higher improvement compared to those on placebo (6 points, *p* = 0.06 for difference). Similarly, there was a tendency for more participants on nortriptyline showing a clinically important response (69%) compared to those on placebo (56%, *p* = 0.08 for difference). The use of other antidepressants has not been examined in knee OA.

Amitriptyline, another older tricyclic antidepressant, has been used to treat neuropathic pain and fibromyalgia [46, 47]. The recent related Cochrane reviews have suggested that although in unselected patients, overall, these do not show efficacy, the proportion of patients showing improvement is higher in the treated group [46, 47]. However, most of these studies did not use an active placebo, and were thus more likely to have been subject to unblinding, as the anticholinergic effects occur at low dose. The authors concluded that although there was not a clinically significant effect in the whole population, a small, but significant proportion of patients do benefit. However further trials were unlikely to be performed. It is not clear which patients are more likely to benefit. We will examine whether the proportion benefiting is higher in those on active treatment, and attempt to identify which are more likely to benefit based on subgroups identified using simple clinical tools (eg severity of pain, radiographic change, painDETECT for pain sensitisation, Pittsburgh sleep quality index for sleep quality, HADS for mood disorders, etc).

This current trial has a number of strengths. It will be the first to examine whether knee pain in OA is effectively treated by low dose amitriptyline, considered by some to be the gold standard for pain sensitisation [17]. It examines low dose amitriptyline against benztropine, an active placebo, reducing the potential for unblinding. It will explore the role of phenotyping, according to relevant clinical features, using simple clinical tools that may be completed by patients and thus could translate into clinical practice. A potential limitation will be that it may be underpowered to perform subgroup analyses.

This study will provide high level evidence regarding the effectiveness of low dose amitriptyline for knee pain in OA compared to benztropine, in reducing pain and improving function. The examination and identification of subgroups more likely to respond, have the potential of informing future research to optimize pain management in knee OA.

## Data Availability

Data sharing is not applicable to this article as no datasets were generated or analysed during the current study.

## Abbreviations

ANCOVA: Analysis of covariance
AQOL-8D: Assessment of quality of life 8D
CONSORT: Consolidated standards of reporting trials
HADS: Hospital anxiety and depression scale
IMMPACT: Initiative on methods, measurement, and pain assessment in clinical trials
IPAQ: International physical activity questionnaire
JSN: Joint space narrowign
KL: Kellgren lawrence
NA: Not applicable
NRS: Numerical rating scale
NSAID: Non-steroidal anti-inflammatory drugs
OA: Osteoarthritis
OARSI: Ostearthritis research society international
PSQI: Pittsburgh sleep quality index
QALYS: Quality adjusted life years
SNRI: Serotonin and norepinephrine reuptake inhibitor
SPIRIT: Standard protocol items: recommendations for interventional trials
TSK: Tampa scale of kinesiophobia
UKU: UKU Side effect rating scale for psychotropic drugs
VAS: Visual analogue scale
WOMAC: Western Ontario and McMaster university arthritis index
